# Preliminary In Vivo Evidence of Oral Selenium Supplementation as a Potentiating Agent on a Vector-Based COVID-19 Vaccine in BALB/c Mice

**DOI:** 10.3390/vaccines11010057

**Published:** 2022-12-26

**Authors:** Muunda Mudenda, Josephine Kimani, Johnson Kinyua, James Kimotho

**Affiliations:** 1 Pan African University Institute for Basic Sciences Technology and Innovation (PAUSTI), P.O. Box 62000, Nairobi 00200, Kenya; 2 Department of Biochemistry, Jomo Kenyatta University of Agriculture and Technology (JKAUT), P.O. Box 62000, Nairobi 00200, Kenya; 3 Innovation and Technology Transfer Department (ITTD), Kenya Medical Research Institute (KEMRI), P.O. Box 54840, Nairobi 00200, Kenya

**Keywords:** severe acute respiratory syndrome coronavirus 2 (SARS-CoV-2), adjuvants, vector vaccine, immuno-modulators, selenium

## Abstract

Evidence of efficacy and toxicity of oral selenium supplementation in vaccine administration against severe acute respiratory syndrome coronavirus 2 (SARS-COV-2) in mice models is scarce. In this study, 4 × 10^9^ virus particles (40 µL) dose of Janssen COVID-19 intramuscular injection vaccine was supplemented with a commercial selenium supplement and sodium selenite orally in BALB/c mice (*N* = 18). Qualitative determination of anti-spike IgG antibody response using indirect Enzyme-Linked Immunosorbent Assay (ELISA) showed significant (*p* ≤ 0.001) increase in anti-spike IgG antibody response for mice groups immunized with vaccine and supplemented selenium. Furthermore, cytokine profiling using real-time quantitative polymerase chain reaction also showed an increase in IL-6 and IL-10 mRNA levels normalized using hypoxanthine phosphoribosyl transferase 1 (Hprt1) and glyceraldehyde 3-phosphate dehydrogenase (Gadph) housekeeping genes. There was no statistical significance (*p* < 0.465) among treated and untreated groups for alanine transaminase (ALT), aspartate transaminase (AST), urea, and creatinine parameters. The study presents preliminary findings and suggests that supplementing Janssen COVID-19 vaccines with selenium can generate more robust immune responses.

## 1. Introduction

The coronavirus disease 2019 (COVID-19) pandemic has led to more than 6.5 million deaths as of 25 September 2022 [1]. While several strategies have been used to manage the pandemic, vaccines have been the most effective [2]. As a result, more than 3 billion people have received at least one dose of COVID-19 vaccine, which has dramatically reduced the disease’s infection rate and severity in most nations [3].

However, the efficacy of available vaccines against various severe acute respiratory syndrome coronavirus 2 (SARS-CoV-2) mutants has been different [4]. Vaccines formulated against COVID-19 using non-replicating viral vector technology have shown comparatively lower efficacy in immunization [5]. Some of the challenges faced with viral vector-based COVID-19 vaccines include the need for multiple doses, poor efficacy against new variants of concern (VOCs) such as delta and omicron, as well as weaker and faster wearing-out immunity [6].

As such, the known potential effect of current viral vector vaccines stands in question, especially as SARS-CoV-2 continues to mutate. Due to these concerns, researchers are constantly improving vaccine technologies in order to be able to offer solutions as COVID-19 progresses, and to keep people protected [7]. Adjuvants can aid in the development of innovative approaches to viral diseases by allowing vaccines to reach their full potential. Consequently, adjuvants are being explored for development of broadly efficacious treatments and vaccines [8].

Oral selenium has attracted considerable attention as a result of its ability to boost the immune system using different mechanisms including stimulating innate immunity of T cells and natural killer (NK) cells [9]. Selenium-supplemented diet has also been found to produce increased titer of antibodies against *Corynebacterium diphtheria* in humans [10,11]. In mice model, deficiency in selenium is reported to decrease the proliferation of T cells while cautious supplementation significantly promoted activation and differentiation of T cells [12]. Furthermore, a mechanistic link has been identified between COVID-19 and selenium, whereby deficiency of selenium is linked to severe progression of COVID-19 while sufficient supplementation of selenium helps to arrest the SARS-CoV-2 viral infection [13,14].

A study reported that, in COVID-19, oral selenium supplementation may be used as supportive therapy for vaccine administration [15]. Another study reported the relationship between oral selenium supplementation and efficacy of a mRNA COVID-19 vaccine in human subjects [16]. However, this study has not been explored for other COVID-19 vaccines such as vector-based vaccines. The current study examined the effectiveness and toxicity of supplementing selenium to the Janssen COVID-19 vaccine in BALB/c mice. The findings of this study will give insight into the application of oral selenium supplements in the administration of vector-based COVID-19 vaccines, whose efficacy has been documented to be low in humans.

## 2. Materials and Methods

### 2.1. Ethical Approvals

Approval was obtained from the Center for Biotechnology Research and Division (CBRD) at the Kenya Medical Research Institute (KEMRI) and the Department of Molecular Biology and Biotechnology at the Pan African University Institute for Basic Sciences Technology and Innovation (PAUSTI). The Mount Kenya University Ethics Review Committee, REF: MKU-ISERC-2398—clearance number 1471, granted permission for the use of mice models. The Janssen vaccine was used in this study solely for research and not for modification or commercialization.

### 2.2. Study Design and Sample Size Determination

This study followed an experimental research study design and was conducted at KEMRI, Innovation & Technology Transfer Division (ITTD). Sample size was determined using the ‘Resource Equation (E)’ for determining minimum and maximum number of animals required in a study [17]. Each experimental group consisted of three mice (*N* = 18). Treatments for the various experimental groups are as shown in Table 1.

### 2.3. Model Animals

Female BALB/c mice (20 ± 2 g, 7–8 weeks old) were used in this study. The mice, purchased from the Institute of Primate Research (IPR)—Kenya, were acclimatized for 7 days at the temperature of 21 ± 3 °C, humidity of 40–70%, 12 h light/dark cycle, and access to appropriate mice chow and water.

### 2.4. Preparation and Administration of Treatments

Sodium selenite (Arichem Limited, Kenya) suspension and the commercial selenium supplement (Troikka Pharmaceutical Limited, Gujarat, India) were prepared freshly every day before treatment in normal saline (0.9% NaCl). Selenium supplements were administered orally using a 25 G gavage for seven (7) days at a dose of 0.04 mg/kg and 0.04 mg/kg by weight for sodium selenite and commercial selenium supplement, respectively [18,19]. The vaccine dose of 4 × 10^9^ viral particles was injected intramuscularly into the left tibialis anterior of experimental BALB/c mice.

### 2.5. Sample Collection

For Enzyme-Linked Immunosorbent Assay (ELISA) and biochemical tests, 50 μL of blood samples were collected on day 14 and 28 by tail puncture in 50 μL of heparin, and centrifuged at 2000 rpm for 10 min. Plasma that had been separated was kept at −20 °C for further examination. Three hundred microliters (300 μL) of whole blood was collected for hematological analysis in 1.5 mL EDTA coated collection tubes using cardiac puncture technique and stored at 8 °C for analysis. Spleen tissues were harvested in sterile 1.5 mL Eppendorf tubes while on ice. Tissues where then stored at −80 °C for downstream experiments. Sacrificing of mice was preceded by euthanization using CO_2_.

### 2.6. Serological Evaluation of SARS-CoV-2 Anti-Spike Protein IgG

Qualitative evaluation of SARS-CoV-2 anti-spike IgG antibody was determined by indirect ELISA using the Mouse Anti-SARS-CoV-2 Spike Protein Antibody IgG Titer Serologic Elisa Kit (Solarbio Science & Technology Co., Ltd., Beijing, China) according to the manufacturer’s instructions. Samples were diluted to a 1:200 ratio dilution using the kit sample diluent. ELISA plates were read at 450 nm using the VersaMax™ ELISA Microplate Reader (Molecular Devices LLC, San Jose, CA, USA).

### 2.7. Extraction of Total RNA from Spleen Tissue

Spleen tissues were homogenized in 1 mL of lysis buffer per 100 mg of sample using copper beads and the Fisherbrand™ Bead Mill 24 homogenizer (Thermo Fisher Scientific, Waltham, MA, USA). Total RNA was extracted using the Total RNA Kit (Solarbio Science & Technology Co., Ltd., Beijing, China) and protocol. The concentration and purity of extracted RNA was checked using the NanoDrop™ 2000/2000c spectrophotometer (Thermo Fisher Scientific, Waltham, MA, USA) at absorbance 260/280 and consequently stored at −80 °C for downstream analysis.

### 2.8. Relative Quantification of IL-6 and IL-10 mRNA Levels

Quantitative Real-Time Polymerase Chain Reaction (qPCR) was performed using the Accuris qMAX One-Step RT-qPCR kits (Accuris Instruments, Edison, NJ, USA) on the Applied Biosystems’ Quant Studio 5 platform (PE Applied Biosystems, Waltham, MA, USA). The experiments used a 25 µL reaction volume and thermo profile as shown in Table 2. The relative gene expression was calculated using the delta-delta thresh-hold cycle (ΔΔCt) formula ΔCt = Ct (gene of interest)—Ct (housekeeping gene) according to [20]. All primer sets used in the study are listed in Table 3.

### 2.9. Haematological and Biochemical Analysis

Hematological analysis was performed using the HumaCount 30^TS^ (Human Diagnostics Worldwide, Wiesbaden, Germany) hematology analyzer machine and protocol. The serum levels of Aspartate Transaminase (AST), Alanine Transferase (ALT), Gamma-Glutamyltransfearse (GGT), urea, and creatinine were determined using the Reflotron colorimetric test kit (Woodley Equipment Company, Lancashire, UK) and protocol.

### 2.10. Data Analysis

Results were expressed as mean of experiments ± standard deviation using Microsoft Excel (2016). Data were plotted using GraphPad prism version 9.2. Unpaired *t*-tests and Analysis of Variance (ANOVA) were performed to compare differences between groups with *p*-value < 0.05 considered significant.

## 3. Results

### 3.1. Qualitative Determination of SARS-CoV-2 Anti-Spike IgG Antibody

#### 3.1.1. Janssen COVID-19 Vaccine Supplemented with Oral Commercial Selenium Supplement and Sodium Selenite Induced Robust Immune Response

**Figure 1:**
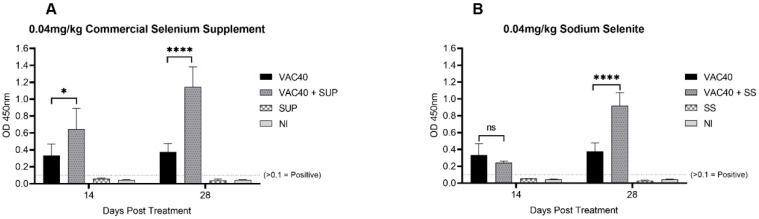
SARS-CoV-2 IgG OD_450nm_ mean values 14 days and 28 days post treatment. (**A**) *t*-tests comparing IgG response for vaccine (VAC40) and vaccine + commercial selenium supplement (VAC40 + SUP). (**B**) *t*-tests comparing IgG response for vaccine (VAC40) and vaccine + sodium selenite (VAC40 + SS). The dashed line indicates the cut-off value for IgG positive OD_450nm_ values. NI = non-immunized group. * Statistical significance (*p* < 0.01). **** Statistical significance (*p* < 0.0001).

Analysis of mean OD_450nm_ values revealed positive response (OD_450nm_ > 0.1) for the vaccine treated groups compared with oral commercial selenium supplement and sodium selenite treated groups alone (Figure 1). The group treated with vaccine and commercial selenium supplement (VAC40 + SUP) showed 1.95-fold increase (*p* < 0.01) in IgG levels compared to the vaccine only group 14 days post treatment (Figure 1A). On the converse, though the IgG levels for the vaccine and sodium selenite (VAC40 + SS) group exceeded the 0.1 OD_450nm_ threshold, the mean OD_450nm_ value was lower (0.73-fold decrease) compared to the vaccine only group 14 days post treatment (Figure 1B). Following statistical analysis with unpaired *t*-tests, samples for groups treated with vaccine and daily diet of supplement both showed significant increase (*p* < 0.0001) in IgG levels 28 days post treatment compared to the vaccine only group (Figure 1A,B).

**Figure 2:**
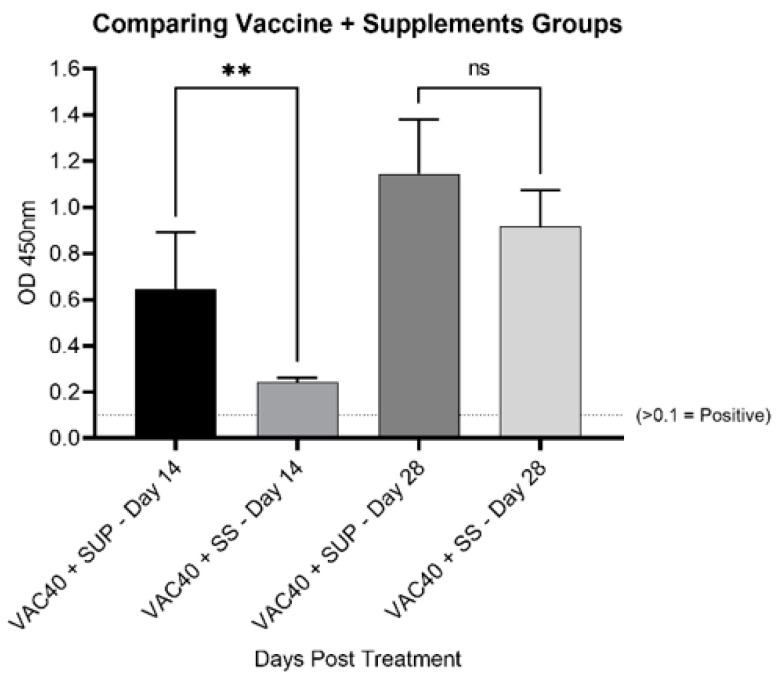
Comparing IgG response for commercial selenium supplement and sodium selenite 14 days and 28 days post treatment. Unpaired *t*-tests to compare the differences for commercial selenium supplement (VAC40 + SUP) and sodium selenite (VAC40 + SS) were performed using GraphPad Prism v.9. The dashed line indicates the cut-off value for IgG positive OD_450nm_ values. ** *p* < 0.001.

Comparison of the IgG levels between commercial selenium supplement (VAC40 + SUP) and sodium selenite (VAC40 + SS) supplemented groups on day 14 revealed significant difference (*p* < 0.002) between VAC40 + SUP and VAC40 + SS with VAC40 + SS showing lower OD_450nm_ (Figure 2). Statistical analysis using unpaired *t*-test for data collected 28 days post treatment showed no significant difference (*p* = 0.076) in IgG levels between VAC40 + SUP and VAC40 + SS (Figure 2). Antibody response analysis showed consistent increase in IgG for both commercial selenium supplement and sodium selenite groups over time (3.04- and 2.44-fold increase, respectively). Overall, mice that received VAC40 + SUP treatment had stronger and more consistent increase in IgG levels 28 days post-treatment compared to VAC40 + SS and VAC40.

#### 3.1.2. Supplementation with Selenium Increased IL-6 and IL-10 mRNA Levels in Mice Models

**Figure 3:**
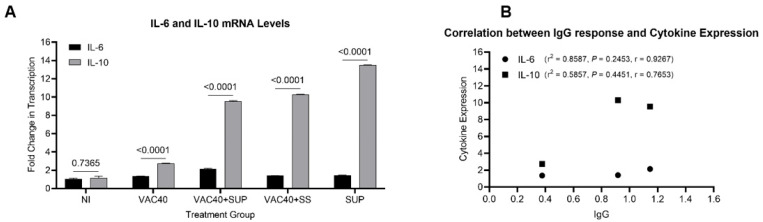
Relative quantification of IL-6 and IL-10 mRNA levels in BALB/c mice. (**A**) Comparison of IL-6 and IL-10 mRNA levels. (**B**) Correlation analysis of SARS-CoV-2 anti-spike IgG and cytokine expression. Unpaired *t*-tests (**A**) and Pearson correlation analysis (**B**) were performed in GraphPad Prism v.9.2 to compare the differences.

Mice immunized with vaccine (VAC40) and daily diet of supplements (VAC40 + SUP, and SUP) produced higher amounts of both IL-6 and IL-10 compared to the vaccine only (VAC40) and non-immunized (NI) groups (Figure 3A). Although there were variances between individual mice, the group treated with commercial selenium supplement (SUP) alone expressed higher amounts of IL-10 compared to both VAC40 + SUP and VAC40 + SS treatment groups. IL-6 mRNA levels were more in the group treated with vaccine and commercial selenium supplement than in any other group. Comparison of IL-6 and IL-10 mRNA levels using a two-factor ANOVA showed significantly higher (*p* < 0.0001) mRNA levels of IL-10 in all treatment (VAC40, VAC40 + SUP, VAC40 + SS, and SUP) groups. There was no significant difference (*p* = 0.7737) in mRNA levels of IL-6 and IL-10 in the non-immunized group. Immunoglobulin G response and cytokine expression were compared and a positive Pearson correlation was observed between IgG and IL-6 (r^2^ = 0.8587), albeit not significant (*p* = 0.2453). Similar observation was made for IL-10 where r^2^ = 0.5857 and *p* = 0.4451 (Figure 3B). Pearson correlation was performed using the OD_450nm_ values and the cytokine fold change values from the treatment groups (VAC40, VAC40 + SUP, and VAC40 + SS).

### 3.2. Toxicological Parameters in BALB/c Mice

#### Cautious Supplementation with Selenium Shows Generally Normal Toxicological Parameters

The analyzed mean values for hematological parameters in various experimental groups are shown in Table 4. Two-way repeated measures ANOVA analysis for the group treated with commercial selenium supplement (VAC40 + SUP) showed significantly (*p* < 0.0001) lower counts of RBC (×10^6^/µL), HGB (g/dL), WBC (×10^3^/µL), HCT (%), and PLT (×10^3^/µL) compared to the non-immunized control group. VAC40, VAC40 + SS, SUP, and SS showed significantly (*p* < 0.0001) more platelet counts compared to the control group. Regarding monocytes, VAC40 + SUP, SUP, and SS showed significantly higher (*p* < 0.0001) values compared to the control group. Other hematological parameters showed no significant toxicological differences (*p* > 0.05) between treated and untreated groups.

**Figure 4:**
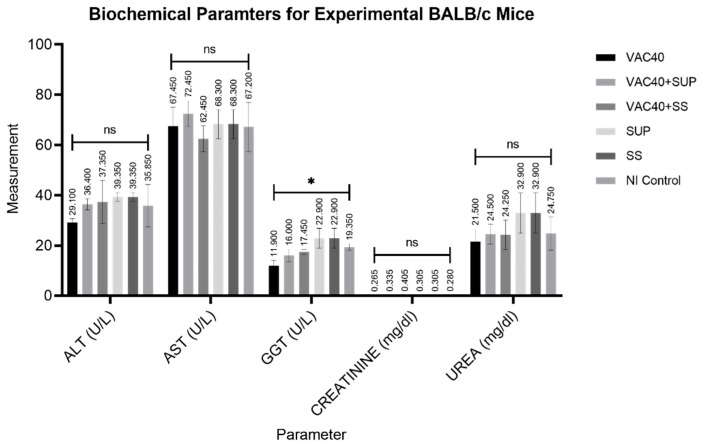
Analyzed results for biochemical parameters of experimental BALB/c mice. VAC40 = vaccine 40 µL, VAC40 + SUP = vaccine 40 µL plus commercial selenium supplement, SUP = commercial selenium supplement, VAC40 + SS = vaccine 40 µL plus sodium selenium, SS = sodium selenite, NI Control = non-immunized group. * Statistical significance (*p* < 0.0408). Analysis of Variance (ANOVA) was performed in GraphPad Prism v.9.2 to compare the differences.

The outcomes of the numerous biochemical tests performed are compiled in Figure 4. Treatment with the vaccine and dietary supplements had no statistically significant effects on the serum levels of ALT, AST, urea, and creatinine (*p* < 0.4507, *p* < 0.4497, *p* < 0.4335, and *p* < 0.1131, respectively). Whereas ONE-WAY ANOVA for GGT did reveal a significant difference (*p* = 0.0408) between experimental groups, Dunnett’s multiple comparison test did not show any statistical differences between the control group and the individual treatment groups (control vs. VAC40, *p* = 0.1123; control vs. VAC40 + SUP, *p* = 0.6444; control vs. VAC40 + SS, *p* = 0.9245; control vs. SUP, *p* = 0.6015; control vs. SS, *p* = 6015).

## 4. Discussion

In this study, IgG response in BALB/c mice was determined serologically using indirect ELISA and the observed higher mean OD_450nm_ values for selenium-supplemented against the non-supplemented groups over the 28-day immunization period support the report that selenium does boost production of anti-spike IgG antibody [24]. However, another study found no correlation between selenium status or intake, and SARS-CoV-2 anti-spike IgG antibody response [16]. The latter was carried out using an mRNA vaccine in human subjects, contrary to that of the present study which was carried out using a vector-based COVID-19 vaccine in BALB/c mice. The variation in the study designs could explain the contradiction in findings between the two studies. Furthermore, comparison of IgG responses between commercial selenium supplement and sodium selenite showed that the commercial supplement elicited stronger and more consistent IgG responses over the 28 days study period. The observation can be explained by studies [16,25], which suggested that supplementation with various micronutrients gives a synergistic effect in boosting the humoral immune response compared to supplementing with sodium selenite only. The commercial selenium supplement also contained Zinc and Vitamin C, two other micronutrients apart from selenium which were recommended in the management of SARS-CoV-2 infection [26].

Another difference observed was the lower mean OD450_nm_ values for VAC40 + SS group compared to the vaccine only group on day 14 (Figure 2). This observation was different from the group treated with vaccine + commercial selenium on the same sampling day (Figure 1A). This observation could be inferred in a study that found that selenium can exhibit both positive and negative immune-modulatory activity depending on dose and duration of exposure [27]. The same study observed that overload of plasma selenium suppresses immune response while sufficient supplementation boosts immune response. Therefore, it is postulated that with passing of days the mice excreted excess selenium in the system, thereby bringing it to healthy levels which then supported immune response. However, this argument can only be supported using a standard plasma selenium concentration test at 14 days and 28 days post treatment [28].

It had been previously reported that vaccination with COVID-19 vaccine induces expression of pro-inflammatory cytokines in mice models [29]. In order to further explore the robustness of immune response induced by Janssen COVID-19 vaccine and supplements in BALB/c mice, the present study determined the splenocyte expression of Interleukin-6 (IL-6) and Interleukin-10 (IL-10) cytokines in experimental groups. It was observed that supplementing the Janssen COVID-19 vaccine (4 × 10^9^ VP) with a commercial selenium supplement (0.04 mg/kg) and sodium selenite (0.04 mg/kg) increased fold change in transcription of IL-10. Although fold change in the transcription of IL-6 was observed, the differences did not reach statistical significance. Interestingly, the commercial selenium supplement showed higher IL-6 and IL-10 than the vaccine treatment alone. The findings are not surprising, as some studies found that selenium supplementation can stimulate the proliferation of inflammatory cytokines, including IL-6 and IL-10 [30,31]. The significance of these findings is supported by a study that reported that a higher presence of inflammatory cytokines was an indicator of higher antibody titers [32]. While this study tried to illustrate this argument using correlation analysis, further exploration is still needed using a larger sample size.

Cytokine storms have been observed in subjects immunized with COVID-19 vaccines [29]. Interleukin-6 (IL-6) and Interleukin-10 (IL-10) have been reported to be important inflammatory cytokines and major characteristics of a SARS-CoV-2 hyper-inflammatory response [32]. IL-6 is a pro-inflammatory cytokine produced by innate immune cells, including natural killer cells, in response to pathogens [32]. IL-10, on the other hand, is secreted by the T helper 2 cells as an immunosuppressive mechanism in hyperinflammation reactions. Consequently, IL-10 is a negative feedback mechanism to suppress acute respiratory distress syndrome (ARDS), damage to vital organs, and severe pneumonia.

In severe cases of SARS-CoV-2 infection, cytokine storms and ARDS lead to tissue damage, multiple organ failure, and, eventually, death [33]. Toxicity of various COVID-19 vaccines is currently under-reported. Furthermore, resulting toxicological effect of Janssen vaccine in BALB/c mice is scarce. As such, the study endeavored to explore the toxicity of Janssen COVID-19 vaccine in BALB/c mice. In this effort, the study evaluated the hematological profiles, liver function, and kidney function of experimental BALB/c mice. Supplementing Janssen COVID-19 vaccine (4 × 10^9^ virus particles) with a commercial selenium supplement (0.04 mg/kg) and sodium selenite (0.04 mg/kg) did not affect the metabolic processes of the experimental BALB/c mice used in the study. Accordingly, the results from this study generally showed normal parameters for ALT, AST, urea, and creatinine. Interestingly, the group treated with vaccine and commercial selenium supplement showed low RBC (×10^6^/µL), HGB (g/dL), WBC (×103/µL), HCT (%), and PLT (×103/µL) counts compared to literature means. However, it is difficult to comment on this abnormality as no literature was found about the same. Considering that severe cases of SARS-CoV-2 infection and COVID-19 vaccination can lead to tissue and organ damage, checking safety alongside efficacy becomes important. However, establishing standard reference ranges for mice hematological and biochemical profiles is a daunting task because of the multiplicity of variables involved. The parameter values can vary depending on the species, age, gender, diet, and the environmental conditions in which the animals are kept. The values can also vary depending on the disease status of the animals.

It is possible to measure the protective effectiveness of antibodies and the success of humoral immune responses following vaccination by measuring the titers of SARS-CoV-2 anti-spike IgG antibodies [34]. Janssen vaccine has showed 72% efficacy in the USA, 66% efficacy in Latin America, and 57% documented efficacy in South Africa [35]. The same study also showed that the efficacy of Janssen vaccine can be boosted to 77% efficacy 14 days post-immunization and 85% after 28 days. The present study demonstrated this hypothesis with observations that showed higher anti-spike IgG, IL-6 and IL-10 levels in mice immunized with vaccine and daily diet of selenium. Furthermore, the safety parameters were generally within acceptable range.

## 5. Conclusions

In conclusion, we observed that oral selenium supplementation could boost the response of SARS-CoV-2 anti-spike IgG antibodies, IL-6, and IL-10 following the immunization with the Janssen COVID-19 vaccine in BALB/c models. Some limitations encountered in the study were as follows: the sample size used during the study was not large enough to offer conclusive results. While the study made interesting findings, only three (3) female BALB/c mice were used per experimental unit, limiting the number of observations that could be made for individual parameters. Since sample size plays a significant role in reaching scientific conclusions, there is a need to repeat the study using a larger sample size. Apart from that, only BALB/c mice were used in the study, leaving questions on whether the same results could be obtained if different species of mice were used. Determining the blood concentration of selenium and using quantitative ELISA assays could give insight into the activity of selenium on the immune system. This study makes it the first to report and provide empirical evidence on the effect of oral selenium supplementation on the Janssen COVID-19 vaccine in BALB/c models.

## Figures and Tables

**Table 1:** Treatment formulations for mice groups.

Group	Treatment Received (3 Mice per Group)
Non-Immunized (NI)	40 μL Normal Saline (0.9% NaCl)
VAC40	40 μL (4 × 10^9^ VP) of Janssen vaccine
VAC40 + SUP	40 μL (4 × 10^9^ VP) of Janssen vaccine + 200 μL daily diet of commercial selenium supplement
VAC40 + SS	40 μL of Janssen vaccine (4 × 10^9^ VP) + 200 μL daily diet of sodium selenite
SUP	200 μL daily diet of commercial selenium supplement (0.04 mg/kg)
SS	200 μL daily diet of sodium selenite (0.04 mg/kg in normal saline)

Abbreviations: VAC40 = vaccine 40 µL, VAC40 + SUP = vaccine 40 µL plus commercial selenium supplement, SUP = commercial selenium supplement, VAC40 + SS = vaccine 40 µL plus sodium selenium, SS = sodium selenite.

**Table 2:** qPCR Reaction Thermo Profile.

Step	Time	Temperature	Cycles
Pre-Denaturation	10 min	95 °C	1
Denaturation	15 s	95 °C	45
Annealing/Extension	1 min	60 °C	

**Table 3:** Summary of reference and target genes used during the study.

Gene	Forward Primer (5′-3′)	Reverse Primer (5′-3′)	Amplicon Size (bp)	Tm (°C)	%GC	NCBI Accession	MGI Sequence ID	Reference
**IL-6**	GAGGATACCACTCCCAACAGACC	AAGTGCATCATCGTTGTTCATACA	141	60	56.52 37.50	XM_021163844.1	96559	[21]
**IL-10**	ATGCCCCAAGCTGAGAACCAAGACCCA	TCTCAAGGGGCTGGGTCAGCTATCCCA	75	60	43.4 50.00	NM_010548.2	96537	[22]
***HPRT1***	TCCTCCTCAGACCGCTTTT	CCTGGTTCATCATCGCTAATC	90	60	52.63 47.62	NM_013556.2	96217	[20]
***GADPH***	TGTCCGTCGTGGATCTGAC	CCTGCTTCACCACCTTCTTG	75	60	57.89 55.00	14433	95640	[20]

Abbreviations: IL-6 = Interleukin 6, IL-10 = Interleukin 10, *HPRT1* = Hypoxanthine Guanine Phosphoribosyl Transferase 1, *GADPH* = Glyceraldehyde-3-phosphate dehydrogenase.

**Table 4:** Hematological parameters for female BALB/c mice immunized with 4 × 10^9^ virus particles doses supplemented with selenium 28 days post-immunization.

PARAMETER	VAC40	VAC40 + SUP	VAC40 + SS	SUP	SS	NI Control	Range
RBC (×10^6^/µL)	7.91 ± 0.01	2.26 ± 0.01 *	8.53 ± 0.01	8.40 ± 0.00	8.60 ± 0.00	7.83 ± 0.12	7.1–9.5
HGB (g/dL)	13.93 ± 0.06	4.10 ± 0.10 *	15.03 ± 0.06	14.53 ± 0.06	15.37 ± 0.06	14.70 ± 0.00	11.6–15.8
HCT (%)	37.47 ± 0.06	10.70 ± 0.00 *	40.70 ± 0.00	38.83 ± 0.06	38.87 ± 0.06	37.37 ± 0.06	37.4–51.7
MCV (fL)	47.27 ± 0.12	47.30 ± 0.10	47.70 ± 0.10	46.13 ± 0.06	45.27 ± 0.06	48.10 ± 0.00	41.5–57.4
MCH (pg)	17.60 ± 0.17	17.50 ± 0.00	17.50 ± 0.00	17.33 ± 0.06	17.77 ± 0.06	18.97 ± 0.06	14.1–18.4
MCHC (g/dL)	37.07 ± 0.06	36.90 ± 0.10	36.90 ± 0.10	37.87 ± 0.06	39.43 ± 0.06	39.30 ± 0.00	30.5–34.2
WBC (×10^3^/µL)	8.53 ± 0.15	1.57 ± 0.06 *	5.07 ± 0.06	5.57 ± 0.15	7.10 ± 0.10	5.60 ± 0.00	-
NEU/SEG (×10^2^/µL)	14.00 ± 1.00	13.67 ± 0.58	8.33 ± 0.58	15.00 ± 0.00	13.00 ± 0.00	9.00 ± 0.00	11–29
LYM (×10^2^/µL)	78.33 ± 0.58	75.33 ± 0.58	86.00 ± 1.00	69.67 ± 0.58	76.67 ± 0.58	86.33 ± 1.15	65–87
MON (×10^2^/µL)	6.97 ± 0.06	11.00 ± 0.00 *	5.33 ± 0.58	14.00 ± 1.00 *	10.00 ± 1.00 *	5.67 ± 0.58	0–6
EOS (×10^2^/µL)	1.00 ± 0.00	1.33 ± 0.58	1.00 ± 0.00	1.33 ± 0.58	0.33 ± 0.58	1.67 ± 0.58	0–5
BOS (×10^2^/µL)	0.00 ± 0.00	0.00 ± 0.00	0.00 ± 0.00	0.00 ± 0.00	0.00 ± 0.00	0.00 ± 0.00	0–1
PLT (×10^3^/µL)	921.33 ± 2.52 *	65.00 ± 0.00*	1110.00 ± 0.00 *	1099.00 ± 1.00 *	1081.67 ± 0.58 *	897.00 ± 1.73	325–888

Abbreviations: VAC40 = vaccine 40 µL, VAC40 + SUP = vaccine 40 µL plus commercial selenium supplement, SUP = commercial selenium supplement, VAC40 + SS = vaccine 40 µL plus sodium selenite, SS = sodium selenite, NI Control = non-immunized control. Range according to [23]. * Statistical significance (*p* < 0.0001). Descriptive statistics were performed in GraphPad Prism v.9.2.

## Data Availability

The data supporting the findings of this study are available upon request from corresponding authors.
